# Reporting of heterogeneity of treatment effect in cohort studies: a review of the literature

**DOI:** 10.1186/s12874-017-0466-6

**Published:** 2018-01-12

**Authors:** Meryl Dahan, Caroline Scemama, Raphael Porcher, David J. Biau

**Affiliations:** INSERM U1153, ECAMO, METHODS, 27 rue du faubourg Saint-Jacques, Université Paris-Descartes, 75014 Paris 5, France

**Keywords:** Cohort studies, Heterogeneity of treatment, Subgroups analysis, Heterogeneity of treatment reporting

## Abstract

**Background:**

This article corresponds to a literature review and analyze how heterogeneity of treatment (HTE) is reported and addressed in cohort studies and to evaluate the use of the different measures to HTE analysis.

**Methods:**

prospective cohort studies, in English language, measuring the effect of a treatment (pharmacological, interventional, or other) published among 119 core clinical journals (defined by the National Library of Medicine) in the last 16 years were selected in the following data source: Medline. One reviewer randomly sampled journal articles with 1: 1 stratification by journal type: high impact journals (the New England Journal of Medicine, JAMA, LANCET, Annals of Internal Medicine, BMJ and Plos Medicine) and low impact journal (the remaining journals) to identify 150 eligible studies. Two reviewers independently and in duplicate used standardized piloted forms to screen study reports for eligibility and to extract data. They also used explicit criteria to determine whether a cohort study reported HTE analysis. Logistic regression was used to examine the association of prespecified study characteristics with reporting versus not reporting of heterogeneity of treatment effect.

**Results:**

One hundred fifty cohort studies were included of which 88 (58%) reported HTE analysis. High impact journals (Odds Ratio: 3.5, 95% CI: 1.78–7.5; *P* < 0.001), pharmacological studies (Odds Ratio: 0.26, 95% CI: 0.13–0.51; *P* < 0.001) and studies published after 2014 (Odds Ratio: 0.5, 95% CI: 0.25–0.97; *P* = 0.004) were associated with more frequent reporting of HTE. 27 (31%) studies which reported HTE used an interaction test.

**Conclusion:**

More than half cohort studies report some measure of heterogeneity of treatment effect. Prospective cohort studies published in high impact journals, with large sample size, or studying a pharmacological treatment are associated with more frequent HTE reporting. The source of funding was not associated with HTE reporting. There is a need for guidelines on how to perform HTE analyses in cohort studies.

**Electronic supplementary material:**

The online version of this article (10.1186/s12874-017-0466-6) contains supplementary material, which is available to authorized users.

## Background

There are different ways to improve patient care. The first one is to develop innovative health-care interventions and this has been the leading contributor for medical improvement so far. The second possibility is to target known, or new, health-care interventions to subgroup of patients where the treatment is more likely to be beneficial. Because patients vary in characteristics such as sex, age, past medical history, genetics, disease severity, presence of comorbidities, concomitant exposures, and other pre-treatment variables, it can be hypothesized that a treatment that has little effect on an unselected group of patients, becomes extremely effective on a specific subgroup of patients. The use of BRAF (BRAF is a human gene that encodes a protein called B-Raf involved in sending signals inside cells that direct cell growth) kinase inhibitor in patients with BRAF mutated metastatic melanoma is one such recent example [1].

Consequently, when reporting on the effect of a treatment, researchers may feel the need to look for subgroup of patients where the effect may differ, either positively or negatively, from that of the other patients. This has been labeled as heterogeneity of treatment effect, namely nonrandom, explainable variability in the direction or magnitude of treatment effects for individuals within a population. Measuring or reporting heterogeneity of treatment effect is widely performed in the randomized trial literature with almost two-third of cardiovascular trials and about one-third of surgical trials reporting such analyses [2]. However, sub-group analyses (a means of assessing heterogeneity of treatment effect) are problematic because they are associated with inflation of type one error, and consequently with reporting of spurious effects, and with poorly controlled type two error, and consequently with a risk for overlooking potential significant heterogeneity [3]. More recently, Sun and colleagues have published an article addressing the issue of measuring and reporting subgroup analyses in randomized controlled trials [4, 5]. They found that almost half randomized trials in a random sample of core clinical journal reported a subgroup analysis with a significant variation in the reporting according to the source of funding, the significance of the main analysis, the sample size, and the impact factor of the journal. Little is known, however, about the measuring and reporting of heterogeneity of treatment effect in cohort studies.

Cohort studies are frequently used to measure the association of a treatment and performing subgroup analyses is likely performed as well. The main advantage of experimental cohort studies over randomized trials is that they usually allow the enrollment of patients who present significant comorbidities or are more fragile, physically, psychologically or socially than those included in randomized designs [6]. Cohort studies have, however, significant drawbacks compared to randomized trials. Mainly, cohort studies are susceptible to selection biases that affect the principal comparison and will also affect any subgroup analyses. This can lead to apparent heterogeneity in treatment effect when there is none or conceal a true difference of effect between categories of patients. Cohort studies are inclined to confounding by indication, therefore subgroups of interest may be identified not because the effect of treatment differs, but because their risk profile differ [7].

Therefore, we decided to perform a review of prospective interventional cohort studies to estimate the proportion of studies reporting a measure of the heterogeneity of treatment effect and identify variables associated with reporting of this heterogeneity.

## Methods

### Protocol and registration

Eligibility criteria, information sources, data items and methods of the analysis were specified in advance and documented in a protocol. Prisma guidelines were followed [8].

### Eligibility criteria

We considered prospective, controlled, cohort studies, measuring the effect of a health care intervention on humans, published in a core clinical journal (as defined by the National Library of Medicine), in the English language. Pharmacokinetic analyses, letters, reviews/meta-analyses, and studies published before 2000 were not considered eligible. The core clinical journals defined by the National Library of medicine, known as the Abridged Index Medicus, included 119 journals in 2015, covering all specialties of clinical medicine and public health sciences [9]. There were no restrictions of participants based on age, sex, socio-economic status, medical condition, associated comorbidities, or other variables. Interventions considered were pharmacological (any treatment where the effect is expected from a drug), interventional (any treatment where the effect is expected from a mechanical cause; for instance a surgical procedure, a rehabilitation program, an angioplasty) or other (any treatment where the effect is expected neither uniquely from a drug nor from a mechanical cause; for instance a psychological intervention, a blood transfusion, a complex intervention encompassing multiple interventions such as a resuscitation method). All types of outcome were considered including time to event, binary and continuous outcomes. The primary outcome was considered for all analyses; in case the primary outcome was not clearly indicated, the first outcome reported in the method section was considered.

### Information sources, search strategy and study selection

Studies were identified by searching Medline via PubMed by two reviewers starting from March 2016 and moving backwards in time until the predefined number of studies was completed. Journals were stratified into high and low impact groups. The six high impact journals were Annals of Internal Medicine [10], British Medical Journal [11], Journal of the American Medical Association (JAMA) [12], Lancet [13], New England Journal of Medicine [14], and Plos Medicine [15]. The low impact journals were the 113 other core clinical journals from the Abridged Index Medicus. The objective was to obtain a total of 150 studies, with 75 in each group. High impact journal studies were identified by generating a random vector of 75 names among the six possible categories; each of the six journals had the same probability of being selected per draw; in case the year 2000 was reached for a journal, the missing number of studies were distributed among other less populated journals. Low impact journal studies were identified by generating a random list of 75 with each journal having a similar probability of being selected per draw; multiple selections (the fact that one journal may contribute to more than one study) were accepted and consequently some core clinical journal would not contribute.

Eligible studies were identified with the following search terms on PubMed « Name of the journal[TA] AND « prospective » AND « cohort study ». The term «Name of the journal[TA]» was replaced by the relevant journal name as randomly selected in the previous step. Hits (*n* = 2019) were reviewed within each journal on title and abstract and then on full text for selection criteria until the adequate number of studies was reached.

### Data collection process

We developed a data extraction sheet, pilot-tested it on ten randomly-selected included studies, and refined it accordingly. Two reviewers (MD, CS) extracted the relevant data from all included studies. Disagreements between the reviewers were resolved by consensus, and if necessary, consultation with an arbitrator (DB). Authors were not contacted for further information. Only the materials and methods section, results section, tables and figures were reviewed; the introduction and discussion sections were not read through; an exception was made for source of funding (see below).

Reviewers also identified a pair-wise comparison of interest, using the following strategy. If there were only two groups, these groups were considered for the analysis. If there were more than two groups, the comparison that was clearly and explicitly defined as the primary comparison in the study report was considered only; if the primary comparison was not explicitly defined, we selected the comparison that reported the largest number of HTE analyses for the selected primary outcome.

### Data items

Heterogeneity of treatment effect was considered if the effect of treatment was reported for all categories of a variable. For instance, if the effect of treatment was reported for men and women separately, or for patients 70 years and older and for those below 70 years old. Heterogeneity of treatment effect was not considered as reported if the effect of treatment was reported for only some of the categories of a variable. For instance, if the effect of treatment was reported for the whole sample under scrutiny, and for men, but not for women. In case an interaction was sought for between a variable and the treatment, and that this interaction was not significant, we considered that heterogeneity of treatment effect was performed even if the effect was not reported among the categories [16].

We extracted information on sample size, length of follow-up, date of publication, funding source, study area, outcome of interest, significance of the effect on the outcome, prespecification of heterogeneity of treatment effect, predictive variables studied. We also looked at whether predictive variables on side effects were reported.

The variable “date of publication” was dichotomized (based on the 0.5 quantile) into studies published between 2000 and 2013 and studies published between 2014 and 2016.

The source of funding was based on statements reported in the method section, disclosure of conflicts of interest, acknowledgments and funding section of the study report. We categorized the source of funding as private, public, mixed, and none; for presentation, we pooled these categories into private (private and mixed) and other. Study areas considered were pharmacological, interventional, and other as described above. The outcome of interest was categorized into time-to-event, binary, and continuous. Prespecification of heterogeneity of treatment effect was considered if the analysis was reported in the method section. Predictive variables were categorized into: age; sex; socioeconomic level (variables referring to income, education, occupation); comorbidities (variables referring to additional diseases or disorders co-occurring with the disease of interest or a measure of comorbidity); severity of the disease under treatment (variables referring to different levels of advancement of the disease of interest); medical history (variables referring to past medical events, previous diseases, or genetic disorders); others; and the form of treatment (variables referring to different doses or different exposure periods or different administration form of the same treatment). Measuring the effect of different forms of treatment can be regarded as not heterogeneity in the effect of treatment but as heterogeneity in the treatment per se. Therefore, HTE studies include those where the form of treatment only was looked at; however descriptive findings are also reported excluding these studies.

Different types of analysis of HTE have been considered: subgroup (presentation of univariate estimate of each category of the variables); adjusted (presentation of estimates adjusted for the other variables); propension (adjustment for selection bias with propensity score methods).

### Risk of bias in individual studies

The Newcastle Ottawa Scale for assessing the quality of non randomized studies was used to rate study quality (Additional file 1). This scale evaluates the quality of the selection and the representativeness of the exposed and non exposed population cohort (four items), the quality of the comparability of cohorts on the basis of the design (one item) and the quality of the assessment of the primary outcome and the adequacy of follow up (three items). Between 0 and 3 total points it was considered as poor quality, between 4 and 6 total points it was considered as moderate quality and between 7 and 8 total points it was considered as good quality of the cohort studies.

### Data analysis

Continuous variables are reported as median and first to third quartile values. Categorical variables are reported as counts and proportion. Descriptive analyses are presented for all studies and for categories of relevant study characteristics such as presence of HTE, journal impact factor, and study area. To examine the association of reporting versus not reporting HTE with study characteristics, we carried out univariable and multivariable logistic regression analyses, with reporting HTE as the dependent variable. The following variables were looked at: sample size, length of follow-up, date of publication (<2014 and ≥2014), funding source (private vs other), study area (pharmacological vs non pharmacological), outcome of interest (time-to-event, binary, continuous), significance of the effect on the outcome (yes vs no), journal impact factor (high vs low impact), quality of the study (continuous), analysis of predictive factors on primary outcome (yes vs no) and their prespecification and analysis of predictive factors on side effects (yes vs no). A multivariable model including predictors with some significance (*P* < 0.2) was then developed. We used the R software version 3.2.2 for all analyses. All comparisons were two tailed, and *P* < 0.05 was considered statistically significant. We used the Wilcoxon rank sum test for the analysis of continuous data and the chi-square/Fisher tests for binary data.

### Additional analyses

A sensitivity analysis excluding studies only looking at the heterogeneity in the form of the treatment from the HTE group was performed. We also performed another sensitivity analysis on looking at the heterogeneity of treatment concerning only the studies considered as good quality of cohort studies, depending on the The Newcastle Ottawa Scale.

## Results

### Study selection

**Fig. 1:**
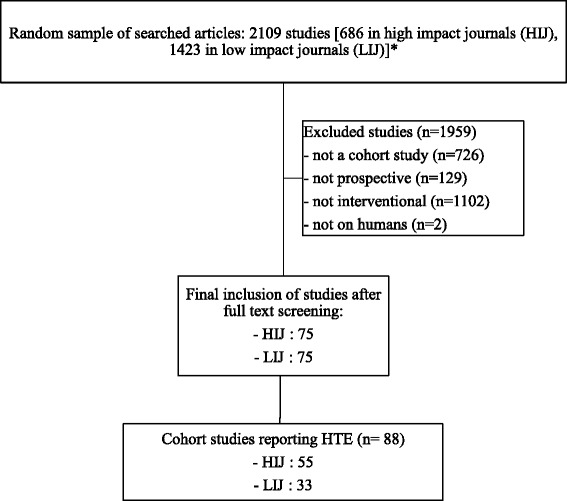
Flow chart of study screening

A total of 150 prospective cohort studies, 75 in high impact and 75 in low impact journals, were identified for inclusion in the review after screening 2019 citations on PubMed (Fig. 1). The year 2000 was reached for the New England Journal of Medicine after 11 cohort studies were retrieved instead of 15 planned and 8 were added to Plos Medicine. Eligible cohorts were identified in low impact journals without the need to correct for numbers.

### Study characteristics

Overall, the median number of patients included was 1633 (Q1 – Q3: 310–9505), 100 (67%) were funded by the industry, and 89 (60%) reported on the effect of a pharmacological treatment (Table 1). Sixty-five (44%) cohort studies addressed time-to-event data, and the primary comparison was significant for 96 (65%) studies. Eighty-eight (59%) studies rated moderately on the Newcastle-Ottawa quality assessment score, and 57 (38%) good.

**Table 1:** Study characteristics in cohort studies reporting and not reporting HTE

Study characteristics	All studies (*n* = 150)	Studies addressing HTE (*n* = 88)	Studies not addressing HTE (*n* = 62)	*p*-value	Studies addressing HTE^a,*^ (*n* = 111)
Sample size, Med(Q1-Q3)	1633(310–9505)	3754(850–20,474)	470(130–2210)	<0.001	1235(201–6133)
Length of follow up (years), Med(Q1-Q3)	3.5(1.2–7.2)	5(2–10)	2(0.5–4.2)	<0.001	3(1–6)
Date of publication				0.04	
2000–2013	72(48)	47(53.4)	23(37)		52(47)
2014–2016	78(52)	41(46.6)	39(63)		59(53)
Journal type, n(%)				<0.001	
High impact factor journals (HIJ)	75(50)	55(62.5)	20(32.2)		52(47)
Low impact factor journals (LIJ)	75(50)	33(37.5)	42(67.8)		59(53)
Source of funding, n(%)				0.85	
Industry	100(66.7)	62(70.5)	38(61.3)		69(62)
Other	31(20.7)	19(21.6)	10(16.1)		22(20)
NA	19(12.6)	7(8)	14(22.6)		20(18)
Study area, n(%)				<0.001	
Interventional	50(32.7)	19(21.6)	31(50)		54(49)
Pharmacological	89(60)	64(72.7)	25(40.3)		56(50)
Other	11(7.3)	5(5.7)	6(9.7)		1(1)
Type of primary outcome, n(%)				0.004	
Time to event	65(44)	48(54.5)	17(27.4)		46(41)
Binary	50(32.7)	24(27.3)	26(42)		37(33)
Continuous	35(22)	16(18.2)	19(30.6)		28(26)
Main effect of primary outcome, n(%)				0.6	
Statistically significant	96(64.6)	58(65.9)	38(61.3)		69(62)
Not statistically significant	54(35.4)	30(34.1)	24(38.7)		42(38)
Newcastle-Ottawa Quality Assessment Score, n(%)				0.82	
0–3	5(3)	3(3)	2(3)		3(3)
4–6	88(59)	54(61)	34(55)		64(58)
7–8	57(38)	31(35)	26(29)		44(40)
HTE reporting	88(58.7)	88(100)	–		49(44)
Analysis of predictive factors on side effects, n(%)	2(1.3)	2(2.3)	–		2(2)
Prespecification of HTE analysis, n(%)	84(56)	84(95.4)	–		46(94)
Type of HTE analysis, n(%)					
Subgroup	2(1.3)	2(2.3)	–		2(4)
Adjusted	67(44.7)	67(76.1)	–		34(69)
Propension	19(12.7)	19(21.6)	–		13(27)
Predictive variables studied, n(%)					
Age	30(20)	30(34)	–		30(61)
Sexe	15(10)	15(17)	–		15(31)
Social level	4(2.7)	4(4.5)	–		4(8)
Genetics	12(8)	12(13.6)	–		12(24)
Treatment	66(44)	66(75)	–		27(55)
Comorbidities	31(20.7)	31(35)	–		31(63)
Severity of disease	13(8.7)	13(14.7)	–		13(26)
Prognostic variables studied, n(%)	51(34)	31(35.2)	20(3.2)	0.73	31(28)
Analysis of prognostic factors on side effect, n(%)	2(1.3)	1(1.1)	1(1.6)	0.71	1(1)
Interaction test, n(%)	27(18)	27(31)	0(0)	–	8(7)

^a^excluding studies assessing only a form of treatment

*to determinate p-value we used the Wilcoxon rank sum test for the analysis of continuous data and the chi square/fisher test for binary data

Eighty-eight (59%) studies reported HTE and 62 (41%) did not (Table 1). Studies reporting HTE had a larger sample size (*P* < 0.001) and length of follow up (P < 0.001), were more likely to be found in high impact journals (P < 0.001), more often assessed a pharmacological treatment (P < 0.001) and addressed time-to-event data (*P* = 0.004). Among the 88 studies reporting on HTE, 84 (95%) pre-specified the analysis in the method section and the most frequent predictive variables evaluated were age (*n* = 30, 34%), comorbidities (*n* = 31, 35%) and treatment (*n* = 66, 75%). Also among the studies reporting HTE, 2(2%) used subgroup analyses, 67(76%) adjusted analysis and 19(22%) propension score methods. An interaction test was performed in 27 studies (18%) [17]. Thirty-nine studies only looked at the heterogeneity of treatment effect in variation of the treatment itself; when excluding these studies from the comparison, there were no differences in the main analysis (Table 1). On multivariable regression analysis, the most important predictors of reporting HTE analysis in the study was the study area (OR: 0.25, 95% CI: 0.09–0.66; *P* = 0.007) with studies evaluating a pharmacological treatment more likely to report HTE (Table 2).

By design, 75 cohort studies were from high impact journals and 75 were from low impact journals. Of the 88 studies reporting HTE, 55 (73%) were reported in high impact journals studies and 33 (27%) in low impact journals; this difference was statistically significant (*P* < 0.001) (Table 3). High impact journals studies were more likely to have a larger sample size (P < 0.001) and a longer follow up (*P* = 0.002) (Table 3). Furthermore, time-to-event data were more frequent in high impact journals (*P* = 0.001) and results more often significant on the primary outcome analysis (*P* = 0.02). When HTE was reported, the pre-specification of HTE analysis in high impact journals studies (*P* = 0.15) and the different type of HTE analysis was similar in both groups; there was no difference in the proportion of variables analyzed for prediction except for medical history (*P* = 0.03).

**Table 2:** Univariate et multivariable regression analysis of factors associated with reporting versus not reporting of HTE analysis

	Logistic univariate regression analysis		Logistic multivariable regression analysis	
Study characteristics	Odds ratio (95% CI)*	*P*-value	Odds ratio (95% CI)*	*P*-value
Sample size	0.99(0.99–1.00)	0.11	1(0.99–1)	0.58
Length of follow up (years)	1.17(1.07–1.30)	0.02	1.1(0.99–1.24)	0.07
Date of publication (<2014 vs ≥2014)	0.5(0.25–0.97)	0.04	0.6(0.16–2.17)	0.42
Journal type (high impact factor)	3.5(1.78–7.5)	<0.001	0.4(0.1–1.55)	0.19
Source of funding(industry funded vs non industry funded)	1 .92(0.82–4.78)	0.85		0.37
Study area (pharmacological vs non pharmacological)	0.26(0.13–0.51)	<0.001	0.25(0.09–0.66)	0.007
Type of primary outcome				
Binary	1(reference)	–	1(reference)	–
Time to event	3.05(1.41–6.80)	0.05	2.22(0.72–7.12)	0.17
Continuous	0.91(0.38–2.17)	0.83	0.98(0.26–3.7)	0.98
Main effect of primary outcome (significant vs non significant)	1.22(0.62–2.40)	0.56	0.97(0.34–2.71)	0.95
Newcastle-Ottawa Quality Assessment Score, n(%) (continuous)				
0–3 4–6 7–8	ref 1[0.13–6.70] 0.79[0.09–5.14]	ref 0.95 0.81	ref 1.61(0.17–1.99) 1.75(0.12–2.3)	ref 0.70 0.66

*in our model, we adjusted on the following variables: source of funding, study area, journal type, main effect of primary outcome, type of primary outcome, quality of the cohort

Eighty-nine cohort studies (59%) evaluated a pharmacological treatment and 61 (41%) did not (Table 4). Pharmacological studies were larger (*P* < 0.001) and reported the analysis of predictive factors more often (*P* < 0.001). Also, there was more industry funded studies in pharmacological studies (*p* = 0.04). The form of treatment was more often analyzed as a predictive variable in pharmacological studies (*p* = 0.002) (Table 4).

**Table 3:** Study characteristics in cohort studies in high impact journals and in low impact journals

Study characteristics	Cohort studies in High impact journals (*n* = 75)	Cohort studies in low impact journals (*n* = 75)	*p*-value*
Sample size, Median(Interquartile range)	4176(1274–23,452)	415(138.5–2586)	<0.001
Length of follow up (years), Median(Interquartile range)	5(2–10)	2.1(1–5)	0.002
Date of publication			<0.001
2000–2013	57(76)	13(17)	
2014–2016	17(24)	62(83)	
Journal type, n(%)			
High impact factor journals (HIJ)	75(100)	–	
Low impact factor journals (LIJ)	–	75(100)	
Study area, n(%)			0.81
Interventional	23(30.7)	27(34)	
Pharmacological	46(61.3)	43(57)	
Other	6(8)	5(9)	
Source of funding, n (%)			0.15
Industry	51(68)	49(65)	
Other	18(24)	12(16)	
NA	6(8)	14(19)	
Type of primary outcome, n (%)			0.001
Time to event	44(58.7)	21(28)	
Binary	19(25.3)	31(41)	
Continuous	12(16)	23(31)	
Main effect of primary outcome, n (%)			0.02
Statistically significant	55(73)	41(54.7)	
Not statistically significant	20(27)	34(45.3)	
Newcastle-Ottawa Quality Assessment Score, n(%)			0.03
0–3	1(2)	4(5)	
4–6	46(61)	42(56)	
7–8	28(37)	29(39)	
HTE reporting	55(73)	33(44)	<0.001
Analysis of predictive factors on side effects, n(%)	2(4)	0(0)	0.53
Prespecification of HTE, n(%)	54(98)	30(91)	0.15
Type of HTE analysis, n(%)			0.44
Subgroup	1(2)	1(3)	
Adjusted	40(73)	27(82)	
Propension	14(25)	5(15)	
Predictive variables studied, n(%)			
Age	23(42)	7(21)	0.06
Sexe	12(22)	3(9)	0.15
Social level	2(4)	2(6)	0.63
Genetics	2(4)	1(3)	0.03
Treatment	44(80)	22(67)	0.21
Comorbidities	23(42)	8(24)	0.11
Severity of disease	9(16)	4(12)	0.76
Prognostic variables studied, n(%)	27(36)	24(73)	0.7
Analysis of prognostic factors on side effect, n(%)	1(1)	1(3)	1
Interaction test, n(%)	21(28)	6(8)	0.06

*to determinate p-value we used the Wilcoxon rank sum test for the analysis of continuous data and the chi square/fisher test for binary data

**Table 4:** study characteristics in pharmacological cohort studies and in non pharmacological cohort studies

Study characteristics	Pharmacological cohort studies (*n* = 89)	Non pharmacological cohort studies (*n* = 61)	*p*-value*
Sample size, Median(Interquartile range)	3434(490–20,482)	1041(151–2714)	<0.001
Length of follow up (years), Median(Interquartile range)	4(1.7–8.3)	3(1–6)	0.14
Date of publication			0.67
2000–2013	44(49)	27(45)	
2014–2016	45(51)	34(55)	
Journal type, n(%)			0.74
High impact factor journals (HIJ)	45(51.1)	29(48)	
Low impact factor journals (LIJ)	44(48.9)	32(52)	
Study area, n(%)			
Non pharmacological	–	61(100)	
Pharmacological	89(100)	–	
Source of funding, n(%)			0.04
Industry	61(68)	39(63)	
Other	15(17)	13(22)	
NA	13(15)	9(15)	
Type of primary outcome, n(%)			0.37
Time to event	42(47)	24(39)	
Binary	30(33)	19(31)	
Continuous	17(20)	18(30)	
Main effect of primary outcome, n(%)			0.55
Statistically significant	53(60)	42(68)	
Not statistically significant	36(40)	19(32)	
Newcastle-Ottawa Quality Assessment Score, n(%)			0.5
0–3	3(4)	2(3)	
4–6	52(58)	36(59)	
7–8	34(38)	23(38)	
Analysis of predictive factors on side effects, n(%)	1(2)	1(2)	0.49
HTE reporting	63(71)	24(39)	<0.001
Prespecification of HTE, n(%)	59(66)	24(100)	0.59
Type of HTE analysis, n(%)			0.40
Subgroup	2(3)	0(0)	
Adjusted	50(79)	16(67)	
Propension	11(18)	8(33)	
Predictive variables studied, n(%)			
Age	19(30)	10(42)	0.19
Sexe	11(17)	4(17)	1
Social level	2(3)	2(8)	0.33
Genetics	9(14)	3(12)	1
Treatment	53(84)	13(54)	0.002
Comorbidities	19(30)	11(46)	0.13
Severity of disease	9(14)	3(12)	0.18
Prognostic variables studied, n(%)	30(48)	21(34)	0.77
Analysis of prognostic factors on side effect, n(%)	1(2)	1(2)	0.52
Interaction test, n(%)	20(22)	7(11)	0.27

*to determinate p-value we used the Wilcoxon rank sum test for the analysis of continuous data and the chi square or fisher test for binary data

**Table 5:** Study characteristics in cohort studies of high quality (7 or 8 on the scale) reporting and not reporting THE

Study characteristics	All studies (*n* = 57)	Studies addressing HTE (*n* = 31)	Studies not addressing HTE (*n* = 26)	*p*-value*
Sample size, Med(Q1-Q3)	2438(415–19,486)	7484(1259–70,336)	470(166–2453)	<0.001
Length of follow up (years), Med(Q1-Q3)	3(1–7)	5(2.2–11.8)	1(0.1–4)	0.002
Date of publication				0.2
2000–2013	28(49)	18(58)	10(38.5)	
2014–2016	29(51)	13(42)	16(61.5)	
Journal type, n(%)				<0.001
High impact factor journals (HIJ)	28(49)	22(71)	6(23)	
Low impact factor journals (LIJ)	29(51)	9(29)	20(77)	
Source of funding, n(%)				0.68
Industry	36(63)	23(74)	13(19)	
Other	10(17)	5(16)	5(50)	
NA	11(20)	3(10)	8(31)	
Study area, n(%)				0.02
Medical	34(60)	23(74)	11(42)	
Non medical	23(40)	8(26)	15(58)	
Type of primary outcome, n(%)				0.05
Time to event	26(46)	18(58)	8(31)	
Binary	16(28)	5(16)	11(42)	
Continuous	15(26)	8(26)	7(27)	
Main effect of primary outcome, n(%)				1
Statistically significant	36(63)	20(65)	16(62)	
Not statistically significant	21(37)	11(35)	10(38)	
Analysis of predictive factors on side effects, n(%)	0(0)	0(0)	–	
Prespecification of HTE analysis, n(%)	30(53)	30(97)	–	
Type of HTE analysis, n(%)				
Subgroup	1(2)	1(3)	–	
Adjusted	23(40)	23(74)	–	
Propension	7(12)	7(23)	–	
Predictive variables studied, n(%)				
Age	13(23)	18(58)	–	
Sexe	7(12)	7(23)	–	
Social level	2(3)	2(6)	–	
Genetics	3(5)	3(10)	–	
Treatment	27(47)	27(87)	–	
Comorbidities	8(14)	8(26)	–	
Severity of disease	6(11)	6(19)	–	
Prognostic variables studied, n(%)	20(35)	10(32)	10()	0.78
Analysis of prognostic factors on side effect, n(%)	0(0)	0(0)	0(0)	–
Interaction test, n(%)	6(10)	6(19)	0(0)	–

*to determinate p-value we used the Wilcoxon rank sum test for the analysis of continuous data and the chi square/fisher test for binary data

### Risk of bias within studies

A total of 88 (59%) studies, 54 (61%) in the HTE group and 34 (55%) in the non HTE group, rated good on the Newcastle Ottawa Quality Assessment Score with no difference between both groups. However, we found that studies reported in high impact journals had a greater quality score than the studies from low impact journals (*P* = 0.03) (table 3). The quality assessment score was not associated with the likelihood of reporting HTE (*P* = 0.82).

### Additional analysis

A sensitivity analysis excluding studies only looking at the heterogeneity in the form of the treatment from the HTE group revealed no relevant differences from the main analyses. It was the same for the analysis about only the good quality cohort studies (Table 5).

## Discussion

Addressing heterogeneity is an interesting method to increase treatment efficiency and decrease unnecessary side effects and health care costs. Heterogeneity is now rather well documented in randomized controlled trials (RCTs) [18–21]. However, cohort studies present different methodological issues which warrant caution when measuring and reporting heterogeneity [22]. Because there was no previous information of how heterogeneity is reported in cohort studies we conducted a review of the literature.

### Summary of evidence

We found that 59% of cohort studies reported HTE. This is significantly more than previously reported for randomized trials [23]. Sun and colleagues in a review of 469 trials found that 44% reported a sub-group analysis [4]. Two possible reasons explaining this difference are wider inclusion criteria and less standardized methodology. Cohort studies are known to include patients at the extreme of selection variables. For instance, cohort studies are more likely to include older patients, patients with more comorbidities, and those with more severe diseases [24]. Therefore, given these patients are included in the cohort, investigators may feel more pressed to analyze the effect of treatment in these particular subgroups [25]. Age, sex, and comorbidities were the most frequent variables assessed for heterogeneity in our study (Table 1). Secondly, the guidelines to conduct and report cohort studies are more recent than those for randomized trials. The CONSORT statement dates back to 1996 [26] when the STROBE guidelines were first published in 2007 [27]. Interestingly cohort studies published before 2014 were more likely to report HTE than those published after 2013 (univariable OR = 0.5, 95% CI: 0.25–0.97; *P* value = 0.04). It could be that researchers, with increased awareness of the methodological issues of HTE analyses, are now being more cautious with subgroup analyses. Factors associated with increased reporting of HTE were cohorts published in a high impact factor journal and cohorts reporting on a pharmacological treatment. The reason for increased reporting of HTE in studies published in high impact journal could be that better studies yield more questions among investigators, reviewers and editors. Accordingly, cohort studies published in high impact journals were more frequently likely to pre-specify HTE analyses in the methods than those published in lower impact journals. It has been previously reported that HTE analyses are more frequently reported in pharmacological treatments [2]. Some issues were found in the use of HTE: analyses were not prespecified frequently enough; selection bias were not accounted for by adequate methods very often (propensity scores) [28].

Contrary to that reported by Sun and colleagues for randomized studies [4], we did not find an association between the source of funding and the reporting of HTE in the cohort. Because cohort studies are not decisional it could well be that sponsors find little incentive in planning, or in performing in retrospect, subgroup analyses [29, 30].

Furthermore, we found that in high impact journals studies, compared to low impact journals studies, there were more analyses of HTE, with more significant result for the primary outcome. The higher rate of reporting HTE in high impact journals may be a result of the independent efforts of investigators. Alternatively, editors and reviewers in high impact journals may be more inclined to request such analyses than those in journals with a lower impact.

Compared to cohorts the main difference lies in the fact that the strength of association between RCTs funding and reporting of subgroup differed in trials with and without statistically significant primary outcomes [31]. In RCTs without statistically significant results for the primary outcome, industry funded trials were more likely to report subgroup analyses (OR: 2.29, CI (95%):1.30 to 4.72) than non-industry funded trials [4]. Industry funded trials were associated with less frequent prespecifCIation of subgroup hypotheses (31.3% v 38.0%, adjusted OR: 0.49, CI (95%):0.26 to 0.94), and less use of the interaction test for analyses of subgroup effects (41.4% v 49.1%, OR:0.52, CI (95%):0.28 to 0.97) than non-industry funded trials [2]. That is not the case for cohort studies. Our results showed that the source of funding and the main effect of primary outcome was not associated with the HTE analysis. These findings further support our hypothesis that RCTs funded by industry are more likely to look for positive subgroup findings when the results are non significant statistically, and suggest that, compared with non-industry funded trials, the quality of carrying out subgroup analyses is more questionable.

### Limitations

Our study has several limitations. First the search terms for identifying a prospective controlled cohort study are quiet brief and this could possible over estimate all eligible studies from the particular journals; this point can likely result in selection bias [32]. We did not search all medical journals and therefore our findings may not be applicable to journals outside our sample. We did, however, include all core clinical journals. Then we dichotomized the journals as high versus low impact and studies as industry funded versus non industry funded. These categorizations ignore both gradients.

One of the limitation is that if journals were randomly selected within their subgroup (low or high impact), studies themselves were identified in a reverse chronological order and constitute a biased sample of all prospective cohort studies within each journal. This bias probably favors a better reporting of heterogeneity in our sample.

Only 150 cohort studies were analyzed. This could be a limitation to provide precise estimates. However, the precision of a 50% probability with 150 trials is +/−8% wich is reasonable.

We did not classify HTE analyses into descriptive or confirmatory as reported by Varadhan R. and colleagues [33]. Although this would have been an interesting theoretical point, it is actually extremely difficult to differentiate between both analyses in practice.

Another limitation is that journal impact factor stands for various other variables such as notoriety, expertise area (although these are general medical journals), submission guidelines, reviewing and editorial process and quality, etc. By comparing low and high impact journal factors we could not identify precisely which variables have an effect on reporting heterogeneity of treatment effect.

## Conclusions

More than 50% of cohort studies published in core clinical journals report some form of heterogeneity of treatment effect analysis. About 20% of cohort studies have adequate methods to account for selection biases. A test for the significance of heterogeneity in the treatment effect is performed in only 18% of cohort studies. Prospective cohort studies published in high impact journals, with larger sample size and longer follow up, studying pharmacological effect of a treatment on a time-to-event primary outcome are associated with more frequent HTE reporting. After multivariable adjustment, the most predictive values associated with reporting HTE were the impact of the journal and the pharmacological study area. Potentially, an important demand will be placed on observational studies to produce evidence to inform decision on some specific subgroup of patients. There is clearly a need for improvement and more clarification of reporting HTE and his analysis [34].

## Additional file

Additional file 1: The Newcastle-Ottawa Quality Assessment Score [35]. (DOCX 11 kb)

## Abbreviations

BMJ: British Medical Journal
BRAF: BRAF is a human gene that encodes a protein called B-Raf involved in sending signals inside cells that direct cell growth
CI: Confident Interval
CONSORT: Consolidated Standards of Reporting Trials
HTE: Heterogeneity of treatment
JAMA: Journal of the American Medical Association
OR: Odds ratio
RCTs: Randomized controlled trials
STROBE: Strengthening the reporting of Observational studies in Epidemiology
VS: Versus
